# Mechanistic Studies of the Negative Epistatic Malaria-protective Interaction Between Sickle Cell Trait and α^+^thalassemia

**DOI:** 10.1016/j.ebiom.2014.10.006

**Published:** 2014-10-13

**Authors:** D. Herbert Opi, Lucy B. Ochola, Metrine Tendwa, Bethsheba R. Siddondo, Harold Ocholla, Harry Fanjo, Ashfaq Ghumra, David J.P. Ferguson, J. Alexandra Rowe, Thomas N. Williams

**Affiliations:** a Kenya Medical Research Institute-Wellcome Trust Research Programme, PO BOX 230-80108 Kilifi, Kenya; b Centre for Immunity, Infection and Evolution, Institute of Immunology and Infection Research, School of Biological Sciences, University of Edinburgh, EH9 3FL, United Kingdom; c Nuffield Department of Clinical Laboratory Science, University of Oxford, John Radcliffe Hospital, OX3 9DU Oxford, United Kingdom; d Department of Medicine, Imperial College, St Mary's Hospital, Praed Street, London W21NY, United Kingdom

**Keywords:** Malaria, Sickle cell trait, α^+^thalassemia, Epistasis, Cytoadhesion, PfEMP1, Red blood cells

## Abstract

**Background:**

Individually, the red blood cell (RBC) polymorphisms sickle cell trait (HbAS) and α^+^thalassemia protect against severe *Plasmodium falciparum* malaria. It has been shown through epidemiological studies that the co-inheritance of both conditions results in a loss of the protection afforded by each, but the biological mechanisms remain unknown.

**Methods:**

We used RBCs from > 300 donors of various HbAS and α^+^thalassemia genotype combinations to study the individual and combinatorial effects of these polymorphisms on a range of putative *P. falciparum* virulence phenotypes in-vitro, using four well-characterized *P. falciparum* laboratory strains. We studied cytoadhesion of parasitized RBCs (pRBCs) to the endothelial receptors CD36 and ICAM1, rosetting of pRBCs with uninfected RBCs, and pRBC surface expression of the parasite-derived adhesion molecule *P. falciparum* erythrocyte membrane protein-1 (PfEMP1).

**Findings:**

We confirmed previous reports that HbAS pRBCs show reduced cytoadhesion, rosetting and PfEMP1 expression levels compared to normal pRBC controls. Furthermore, we found that co-inheritance of HbAS with α^+^thalassemia consistently reversed these effects, such that pRBCs of mixed genotype showed levels of cytoadhesion, rosetting and PfEMP1 expression that were indistinguishable from those seen in normal pRBCs. However, pRBCs with α^+^thalassemia alone showed parasite strain-specific effects on adhesion, and no consistent reduction in PfEMP1 expression.

**Interpretation:**

Our data support the hypothesis that the negative epistasis between HbAS and α^+^thalassemia observed in epidemiological studies might be explained by host genotype-specific changes in the pRBC-adhesion properties that contribute to parasite sequestration and disease pathogenesis in vivo. The mechanism by which α^+^thalassemia on its own protects against severe malaria remains unresolved.

## 1 Introduction

The burden of malaria, currently estimated at over 2 million episodes of clinical disease and 655,000 deaths annually (WHO, 2012), has exerted strong selection on the human genome, leading to the occurrence at high frequencies of a number of host-protective polymorphisms (Kwiatkowski, 2005). Some of the best-documented examples are inherited disorders of hemoglobin that include sickle hemoglobin (HbS) and α^+^thalassemia (Williams, 2006). Carriers of HbS, which results from the substitution of valine for the usual glutamic acid at the 6th position of the β-globin amino acid chain, have sickle cell trait (HbAS), a clinically silent condition that is associated with a high degree of protection against all forms of clinical malaria (Taylor et al., 2012, Williams et al., 2005a). α^+^thalassemia, which results from a deletion of one of the paired α-globin genes on chromosome 16 (− α), has been shown to protect against severe and fatal malaria in both its heterozygous (− α/αα) and homozygous (− α/− α) forms (Taylor et al., 2012, Williams et al., 2005b). The significant overlap in the geographic distributions of these two conditions means that they are frequently co-inherited (Flint et al., 1998). Nevertheless, rather than conferring an additive advantage, co-inheritance of both HbAS and α^+^thalassemia is associated with the loss of the malaria-protection that is afforded by each polymorphism individually (Williams et al., 2005c, Crompton et al., 2008, May et al., 2007), through an unknown mechanism.

Sequestration of mature-stage *Plasmodium falciparum*-infected RBCs (pRBCs) in the deep microvasculature appears to be central to the pathogenesis of severe malaria (Miller et al., 2002, van der Heyde et al., 2006). This process involves both the binding of pRBCs directly to the vascular endothelium (cytoadhesion) and the binding of pRBCs to uninfected RBCs to form cell clusters known as rosettes (Rowe et al., 2009). Both *P. falciparum* cytoadhesion and rosetting are mediated by *P. falciparum* erythrocyte membrane protein-1 (PfEMP1), a parasite-encoded protein that is expressed on the surface of mature-stage pRBCs (Rowe et al., 1997, Baruch et al., 1996). Individually, it has been suggested that both HbAS and α^+^thalassemia may protect against severe malaria via reduced cytoadhesion and rosetting, and that this reduction might be mediated by reduced expression of PfEMP1 on the pRBC surface (Carlson et al., 1994, Udomsangpetch et al., 1993a, Udomsangpetch et al., 1993b, Cholera et al., 2008, Krause et al., 2012, Butthep et al., 2006). Nevertheless, the literature regarding α^+^thalassemia is not completely consistent in this regard, with no effect on cytoadhesion having been found in two previous studies (Williams et al., 2002, Luzzi et al., 1991a) and others having reported equal or raised levels of pRBC surface antigen expression (potentially due to raised PfEMP1 expression levels) (Williams et al., 2002, Luzzi et al., 1991b). We were therefore interested to investigate whether the negative epistatic interaction between HbAS and α^+^thalassemia might relate to changes in cytoadhesion, rosetting and PfEMP1 expression in pRBCs of mixed genotype, a hypothesis that we investigated in the current study using the largest panel of variant RBCs studied to date.

## 2 Materials and Methods

### 2.1 Red Blood Cells

The static adhesion, rosetting and PfEMP1 experiments were conducted using RBC samples selected on the basis of Hb phenotype and α^+^thalassemia genotype, collected from members of a cohort of children aged between 6 months and 11 years involved in a study of the immune-epidemiology of malaria in Kilifi County on the coast of Kenya (Williams et al., 2005a, Wambua et al., 2006). Samples were collected during cross-sectional surveys conducted in May 2009 and May 2010. Whole blood samples were collected into heparinized tubes and screened for malaria both by using a rapid diagnostic test (OptiMAL® Diamed, Morat, Switzerland) and by light microscopy of thick and thin Giemsa-stained blood films. Only blood samples from children testing negative by both methods were used. Whole blood was pelleted by centrifugation before removing the plasma by aspiration and then the white blood cells by density centrifugation through Lymphoprep™ (Fresenius Kabi Norge AS for Axis-Shield PoC AS, Oslo, Norway). Purified RBC pellets were washed twice then resuspended at 50% hematocrit in RPMI 1640 medium (Invitrogen) supplemented with 25 mM HEPES, 2 mM l-glutamine (Invitrogen), 25 μg/ml gentamicin, 20 mM d-glucose (Sigma) and 6 mM NaOH (incomplete RPMI). For cytoadhesion assays, RBCs were stored at 4 °C and used within 4 days of collection. For rosetting and flow cytometry experiments, RBCs were cryopreserved in glycerolyte within 24 h of collection and thawed by standard methods (Kinyanjui et al., 2004, Deans et al., 2006) the day before an experiment.

The proportion of HbS in RBCs collected from HbAS individuals of various α^+^thalassemia genotype was determined in members of a second cohort study of children, the Kilifi Genetic Birth Cohort (KGBC) study (Williams et al., 2009), from whom capillary samples of whole blood were collected at enrolment (at 9–12 months of age) into EDTA anticoagulant (Becton Dickinson, NJ, USA) between February 2006 and April 2009.

### 2.2 DNA Extraction and Genotyping

DNA was extracted either from fresh whole blood samples using the semi-automated ABI PRISM® 6100 Nucleic acid prep station (Applied Biosystems) or from EDTA blood samples previously stored at − 80 °C using the QIAamp DNA Blood Mini Kit (Qiagen). Genotyping at the *HBB* locus to distinguish between HbAA and HbAS individuals and at the *HBA* locus for the 3.7 kb α-globin deletion that gives rise to common African variant of α^+^thalassemia was conducted by PCR as described in detail previously (Waterfall and Cobb, 2001, Chong et al., 2000). Swain-Langley (Sl) and McCoy (McC) Complement Receptor One (CR1) Knops blood group genotypes were determined using the Sequenom iPLEX® Gold MassArray SNP genotyping platform as previously described (Fry et al., 2008).

### 2.3 RBC Phenotyping

ABO blood group phenotypes were determined by hemagglutination (Alba Bioscience, Edinburgh, UK). RBC surface CR1 expression levels were determined by flow cytometry as previously described (Cockburn et al., 2002). The proportion of intracellular HbS in RBCs from HbAS individuals was determined in fresh samples by high performance liquid chromatography (HPLC) (Variant Analyzer, Bio-Rad, Hercules, CA, USA) using the β-thalassemia short program (Williams et al., 2009). Full blood counts for the determination of RBC mean cell volumes were performed on fresh EDTA-anticoagulated blood samples using an MDII-18 counter (Beckman-Coulter, Fullerton CA, USA).

### 2.4 Parasites and Parasite Culture

*P. falciparum* ItG parasites (referred to as ItG-ICAM in some earlier studies), a strain that binds to both CD36- and ICAM1 (Baruch et al., 1996, Adams et al., 2000), was used for cytoadhesion assays, and IT/R29 (Roberts et al., 1992) and TM284R + (Scholander et al., 1996) were used for rosetting assays. ItG and IT/R29 have the same genotype but have been selected for different adhesion phenotypes and express different PfEMP1 variants (Rowe et al., 1997, Springer et al., 2004). Parasites were cultured as described previously (Deans and Rowe, 2006) in blood group O^+^ human RBCs at 2% hematocrit in supplemented RPMI (as described above) plus 10% pooled non-malaria-exposed human serum. ItG parasites were selected for binding to ICAM1 and aliquots of the selected parasites cryo-preserved. Thawed aliquots were returned to culture and used within three weeks of thawing. The rosetting IT/R29 and TM284R + parasite strains were maintained at baseline rosetting frequencies of > 50% by repeated selection using gelatin flotation or density centrifugation on 60% Percoll (Sigma) as previously described (Handunnetti et al., 1992). TM284R + parasites showed lower rosette frequencies (30–40%) when grown in 96 well plates during experiments.

### 2.5 Magnetic Selection and Invasion

RBCs infected with late-stage pigmented trophozoites were purified to > 90% parasitemia from uninfected and ring-stage infected RBCs by magnetic-activated cell sorting (MACS®) as described in detail previously (Ribaut et al., 2008, Uhlemann et al., 2000). In the case of the rosetting parasite strains IT/R29 and TM284R +, buffer preparations were supplemented with fucoidan at a final concentration of 50 μg/ml to prevent rosette formation in culture (Kyriacou et al., 2007). α^+^thalassemic RBCs display dose-dependent reductions in mean cell volume (MCV) and mean cell hemoglobin (MCH) and a dose-dependent increase in erythrocyte count (Williams et al., 1996). In order to ensure comparable starting parasitemias in α^+^thalassemic RBCs compared to normal RBCs, RBC counts for each genotype were determined using a Neubauer improved hemocytometer (BlauBrand, Wertheim, Germany). Purified trophozoites were then used to invade the test RBCs at a starting parasitemia of 3.0% (approximately 2.88 × 10^5^ pRBCs and 9.6 × 10^6^ uninfected test RBCs), except for the ItG binding assays when the cultures were initiated at a parasitemia of 1.5%. Parasites were cultured in 96-well flat-bottomed plates (100 μl volume) (Corning Costar Co., Cambridge, MA, USA) or small flasks (10 ml volume) at 2% hematocrit for one complete life-cycle to mature pigmented trophozoite stages and then used in adhesion assays, rosetting assays or flow cytometry.

### 2.6 Static Adhesion Assays

Binding assays were performed as described in detail previously (Newbold et al., 1997, Ochola et al., 2011) using the purified recombinant proteins CD36 (R & D Systems, UK) and ICAM1-Fc (a gift from Professor Alister Craig, Liverpool School of Tropical Medicine). Bacteriological Petri dishes (Falcon 1007; Becton Dickinson, Oxford, UK) were coated with proteins at a concentration of 50 μg/ml at 37 °C for 2 h and then blocked with Phosphate buffered saline (PBS)/1% BSA overnight at 4 °C with an additional incubation at 37 °C for an hour. Each Petri dish was then incubated for 1 h at 37 °C with a 1.25 ml parasite suspension at 1% hematocrit and 3% mature pigmented trophozoites in each donor's specific RBCs. Each donor RBC sample was tested in duplicate Petri dishes. Bound infected RBCs were fixed with PBS/1% glutaraldehyde for 1 h at 4 °C before staining with 10% Giemsa for 15 min. All wash steps and cell suspensions were conducted in bicarbonate-free RPMI 1640 in 2% glucose, adjusted to pH 7.2. Images of adherent RBCs were captured using an inverted microscope with six images taken across random fields for each of triplicate spots for each protein on each plate, giving 36 images per protein for each RBC donor. Image processing and analysis was carried out using Image SXM software and the results expressed as the mean number of parasitized erythrocytes bound per mm^2^ of surface area (Barrett, 2008, Paton et al., 2011). It was not technically feasible to perform cytoadherence assays in all 99 RBC samples on the same day, so the experiments were carried out on 5 separate days. Each sample was tested in two dishes run on the same day and with triplicate spots of each protein in each dish. Every experimental day included at least one sample of normal genotype RBCs (AA αα/αα).

### 2.7 Rosetting Assays

Rosette frequency was assessed by fluorescence microscopy of ethidium-bromide stained culture suspension as previously described (Deans and Rowe, 2006). Rosette frequency was defined as the percentage of mature pigmented trophozoite-stage pRBCs binding two or more uninfected RBCs among every 200 pRBCs counted. For each sample, rosette frequency was counted from duplicate wells and the mean rosette frequency determined. We investigated rosetting in IT/R29 parasites in a total of 59 RBC samples over two successive experimental days (day 1 n = 30 and day 2 n = 29). Rosetting in TM284R + parasites was tested in a total of 91 RBC samples in three separate experiments divided by blood group, O (n = 31), A (n = 29) and B and AB (n = 31). Each of the three TM284R + rosetting experiments was repeated and the mean rosette frequency from the duplicate experiments determined.

### 2.8 Flow Cytometry

PfEMP1 expression in live ItG pRBCs was tested on a single experimental day in a total of 60 RBC samples. A three-step staining process was used, with a 1:50 dilution of rat polyclonal anti-serum raised against the ITvar16 PfEMP1 variant (a gift from Professor Alister Craig), a secondary 1:33 dilution with goat anti-rat IgG (Serotec) and a tertiary 1:250 dilution with Alexa Fluor 488 conjugated donkey anti-goat IgG (H + L) (Invitrogen) plus 1.25 μg/ml of Hoechst 33342 (Invitrogen). All incubations were for 1 h on ice, followed by three washes in PBS. A negative control incubated with The full name Phosphate buffered saline moves to line 204 PBS in place of the primary antibody was included for each test sample, with secondary and tertiary antibodies as above. Samples were fixed in 0.5% paraformaldehyde prior to acquisition. For each sample, a total of 200,000 events were acquired on an LSR II flow cytometer (Becton Dickinson Biosciences) and analyzed using proprietary software (Flow-Jo V9, Treestar Inc., Ashland, OR, USA). Due to low specific antibody binding, pRBCs stained with the ITvar16 PfEMP1 antisera resulted in a shift in the pRBC population when compared to the negative control samples, rather than distinct antibody-positive and antibody-negative populations (Fig. S1). The whole Hoechst positive pRBC population was therefore gated, and the Alexa Fluor 488 median fluorescent intensity (MFI) was measured. For each donor the MFI of the negative control (Fig. S1A) was subtracted from the MFI of the stained sample (Fig. S1B) to determine the ITvar16-specific MFI.

Flow cytometry to measure PfEMP1 RBC surface expression in the rosetting parasite strains IT/R29 and TM284R + was carried out on the same days and using the same samples as the rosetting assays. Staining was as previously described (Ghumra et al., 2012) with 10 μg/ml of anti-ITvar9 (Ghumra et al., 2011) or anti-TM284var1 (Ghumra et al., 2012) rabbit polyclonal total IgG respectively, followed by Alexa Fluor 488 conjugated goat anti-rabbit IgG (H + L) (Invitrogen) at 1:1000 dilution. Fucoidan at a final concentration of 50 μg/ml was used throughout the staining and acquisition steps to disrupt rosettes. Otherwise, the incubation, washing, fixation and acquisition were as above. ITvar9 or TM284var1 specific MFI was determined by taking the MFI of pRBCs positively staining for ITvar9 or TM284var1 PfEMP1 variants (upper right quadrant) and subtracting the MFI of uninfected RBC samples (lower left quadrant) (Fig. S2). For the determination of ITvar9- or TM284var1-specific positive pRBC proportions, the proportion of pRBCs positively staining (upper right quadrant) with the negative control (10 μg/ml of total IgG from a non-immunized rabbit; Fig. S2A) was subtracted from the proportion of pRBCs positively staining with the specific antibody (Fig. S2B).

### 2.9 Statistical Analysis

All adhesion, rosetting and flow cytometry experiments were carried out blinded to RBC genotype. All statistical analyses were conducted using STATA v11 (StataCorp LP, Texas, USA) and graphs generated using Graph Pad Prism v5 (GraphPad Software Inc, San Diego, CA, USA). The effect of HbAS and α^+^thalassemia on static adhesion, rosetting and PfEMP1 expression was tested in a multivariate linear regression model, including experimental day as a covariate to account for day-to-day variation in assay performance. Additional confounding variables previously suggested to affect parasite adhesion phenotype were examined for inclusion in the model, including host ABO blood group (Udomsangpetch et al., 1993b, Carlson and Wahlgren, 1992, Rowe et al., 1995, al-Yaman et al., 1995, Chotivanich et al., 1998), *Sl* and *McC* Knops blood group genotype (Rowe et al., 1997), RBC surface CR1 expression level (Rowe et al., 1997) and RBC mean cell volume (MCV) (Carlson et al., 1994). Inclusion of variables in the multivariate model was based on a univariate analysis, with any variable displaying P < 0.05 being tested for inclusion in the multivariate model. The final model used for each analysis was determined by examining the effect of each variable on the model fit using the log-likelihood ratio (lr) test. Only variables that improved model fit were kept in the final analyses, and these are indicated in the figure legends for each experiment. All non-normally distributed data were normalized by square-root transformation prior to analysis, and the resulting coefficients and 95% confidence intervals were transformed back to the original scale to display graphically. A two-sided significance level of < 0.05 was considered statistically significant for all analyses. We tested for evidence that a reversal in static adhesion, rosetting and PfEMP1 expression with co-inheritance of HbAS and α^+^thalassemia is the result of an interaction between HbAS and α^+^thalassemia using the likelihood ratio (lr) test for interaction.

As an alternative way of presenting the data, we also show in the Supplementary figures the individual data points for each donor, adjusted to account for day-to-day variation in assay performance by normalizing the data from each sample to the mean of the control (HbAAαα/αα) cells run on the same day. Differences between median binding values for each genotype were assessed by the Kruskal–Wallis test with Dunn's multiple comparisons post-hoc test. This approach has the benefit of visualizing the variation between donors within each genotype more clearly than the figures in the main text. However, the linear regression model provides the most appropriate statistical analysis of the data. Both methods gave similar results.

### 2.10 Ethical Conduct of Research

All blood samples were collected following individual informed written consent from participants or their legal guardians. The study was approved by the Kenya Medical Research Institute National Ethical Review Committee in Nairobi and was conducted in accordance with the Declaration of Helsinki.

## 3 Results

### 3.1 Independently, Both α^+^thalassemia and HbAS are Associated With Reduced Cytoadhesion, a Phenomenon That is Reversed When Both Polymorphisms are Inherited Together

Individually, both HbAS and α^+^thalassemia have been associated with reduced *P. falciparum* cytoadhesion in some studies (Cholera et al., 2008, Krause et al., 2012, Butthep et al., 2006); however, no data are available regarding the effect of their co-inheritance. We investigated the effect of co-inherited HbAS and α^+^thalassemia on cytoadhesion of pRBCs infected with parasites of the ItG strain (which binds to both CD36 and ICAM1 (Baruch et al., 1996)) in a total of 99 RBC samples of various Hb and α^+^thalassemia genotype combinations. Purified ItG pRBCs were invaded into donor RBCs and grown for one asexual cycle before testing. The experiments were performed across five separate days, so we analyzed the data by linear regression and included experimental day as a covariate in the model. Alternative presentations of the data, showing individual data points and adjusting for day-to-day binding variation by normalizing each sample to the mean binding of the control RBC samples (AA αα/αα) for each day, are shown in the supplementary figures.

**Fig. 1:**
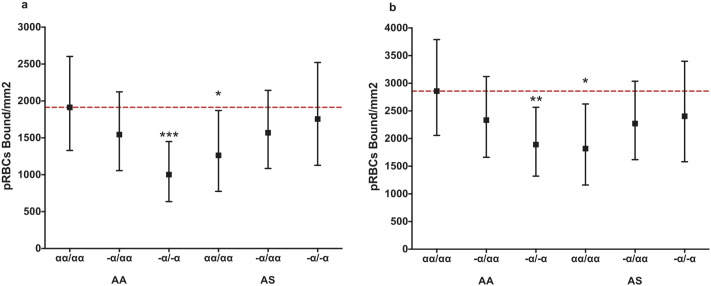
Cytoadherence of *P. falciparum* ItG pRBCs by HbAS and α^+^thalassemia genotype. Binding to (A) CD36 and (B) ICAM1 recombinant proteins. Static adhesion was investigated in a total of 99 RBC samples representing the six possible HbAS and α^+^thalassemia genotype combinations (AA αα/αα (N = 21), AA − α/αα (N = 18), AA − α/− α (N = 21), AS αα/αα (N = 11), AS − α/αα (N = 18), AS − α/− α (N = 10)) using the *P. falciparum* ItG parasite strain. Data are expressed as the mean number of parasitized erythrocytes bound per mm^2^ (95% confidence intervals) as derived by linear regression. The dotted line shows the mean binding in the control (AA αα/αα) pRBCs. Adhesion for each sample was tested in 2 dishes with triplicate protein spots in each dish. Values that are statistically significantly different to the wild type value by linear regression are in asterisk *P = 0.05 to 0.01, **P < 0.01 to 0.001 and ***P < 0.001.

In agreement with observations from one previous study (Krause et al., 2012) but in contrast to those from two others (Williams et al., 2002, Luzzi et al., 1991a), we found that α^+^thalassemia was associated with reduced binding to CD36, an observation that was most marked in homozygotes (mean binding 1001.7 parasites/mm^2^ [95% CI 635.9–1450.2] compared to 1913.4 [1329.1–2603.9] in HbAAαα/αα controls (P < 0.001)) (Fig. 1A, S3A & Table S1). HbAS alone was also associated with significantly reduced binding to CD36 (1263.1; 774.3–1870.8; P = 0.037). However, the adhesion in HbAS pRBCs increased sequentially with α^+^thalassemia deletions, such that no significant reduction was seen in pRBCs with both HbAS and either heterozygous or homozygous α^+^thalassemia (likelihood ratio (lr) test for interaction χ^2^ = 11.14, P = 0.004).

Patterns of pRBC binding to ICAM1 were similar to those seen for CD36, with homozygous α^+^thalassemia being associated with significantly lower pRBC binding (1891.9; 1320.5–2565.7) in comparison to normal pRBCs (2858.2; 2057.1–3790.8; P = 0.007) (Figs. 1, S3 & Table S1). Binding to ICAM1 was also lower in HbAS non-thalassemia pRBCs (1819.2, 1160.7–2625.1; P = 0.014) (Figs. 1, S3 & Table S1). However, as for CD36, these effects were reversed in pRBCs with co-inherited HbAS and α^+^thalassemia (lr test for interaction χ^2^ = 7.50, P = 0.024).

### 3.2 Altered PfEMP1 Expression Correlates With Reduced Cytoadherence in HbAS but not in α^+^thalassemic pRBCs

**Fig. 2:**
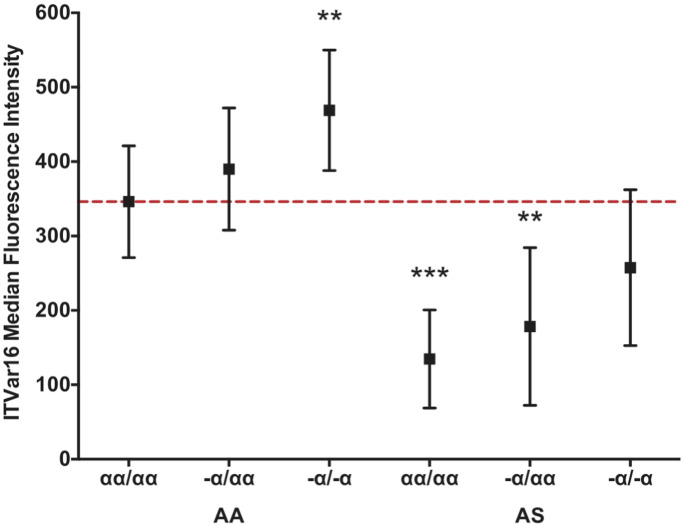
PfEMP1 expression in *P. falciparum* ItG pRBCs by HbAS and α^+^thalassemia genotype. PfEMP1 expression by the ItG parasite strain was determined by flow cytometry using rat polyclonal antisera raised against the ITvar16 PfEMP1 variant predominantly expressed by the ItG parasite strain. A total of 60 RBC samples representing the HbAS-α^+^thalassemia genotype combinations were tested (AA αα/αα (N = 10), AA − α/αα (N = 10), AA − α/− α (N = 11), AS αα/αα (N = 10), AS − α/αα (N = 9), AS − α/− α (N = 10)). Differences in PfEMP1 expression by genotype were analyzed by linear regression with adjustment for confounding by the ABO blood group. Results are expressed as the mean MFI (95% CI). The dotted line shows the mean MFI in the control (AA αα/αα) pRBCs. MFI for each sample was tested in duplicate.

PfEMP1 is the parasite-derived molecule expressed on the mature-stage pRBC surface that mediates cytoadherence to both CD36 and ICAM1 (Baruch et al., 1996, Springer et al., 2004). Previous studies have implicated reduced PfEMP1 expression as a mechanism for the reduced cytoadherence that is associated with HbAS (Cholera et al., 2008), although the data for α^+^thalassemia have been inconclusive (Krause et al., 2012, Williams et al., 2002, Luzzi et al., 1991b). We were therefore interested to determine whether the genotype-specific patterns of binding we noted in our cytoadherence experiments might be explained by differences in PfEMP1 expression. We used magnetically purified mature-stage ItG-strain parasites to invade 60 RBC samples of assorted HbAS and α^+^thalassemia genotype combinations. Consistent with data from one previous study (Cholera et al., 2008), we found that compared to normal pRBCs (Median Fluorescent Intensity (MFI), 346.2 (271.0–421.3), PfEMP1 expression was significantly lower in HbAS pRBCs in the absence of α^+^thalassemia (134.6; 68.7–200.5; P < 0.001) (Figs. 2, S4 & Table S1). However, in contrast to another previous report (Krause et al., 2012) but consistent with data from two others (Williams et al., 2002, Luzzi et al., 1991b), α^+^thalassemia alone was associated with increased expression, of PfEMP1, an observation that was significant for the homozygous genotype (469.0; 388.0–549.9; P = 0.004). The reduced PfEMP1 expression associated with HbAS was reversed with co-inheritance of α^+^thalassemia (Figs. 2, S4 & Table S1), although the interaction test was not significant (lr test χ^2^ = 0.47, P = 0.789). While the decreased surface area of α^+^thalassemic RBCs may act as a potential confounder in these experiments (Luzzi et al., 1991a, Luzzi et al., 1991b), adjusting for cell surface area made no material difference to the interpretation of these data (not shown).

### 3.3 Reduced Rosette Frequency and PfEMP1 Rosetting Variant Expression in HbAS pRBCs is Reversed on Co-inheritance With α^+^thalassemia

**Fig. 3:**
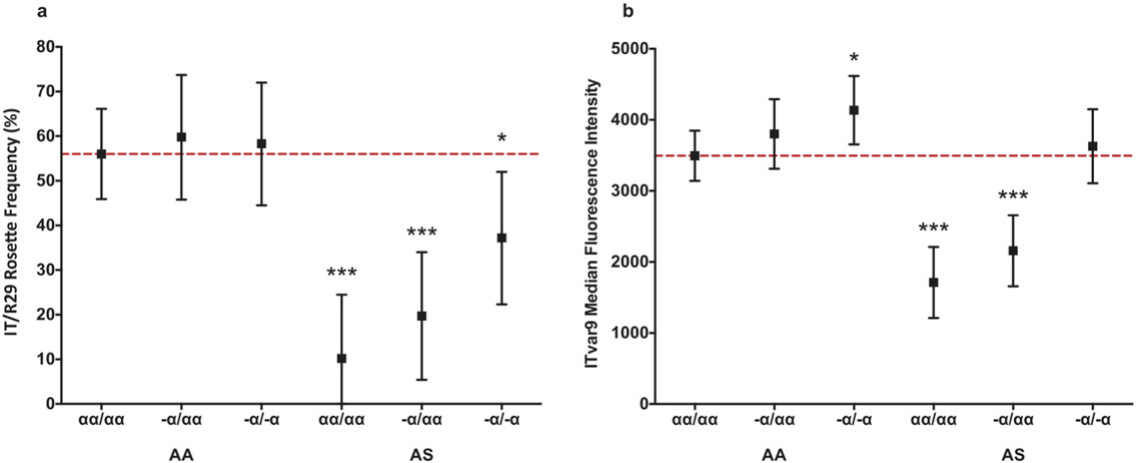
Rosette frequency and PfEMP1 expression in *P. falciparum* IT/R29 pRBCs by HbAS and α^+^thalassemia genotype. (A) IT/R29 rosette frequency. (B) ITvar9 PfEMP1 expression. Rosetting and PfEMP1 expression in the IT/R29 parasite strain were assessed on a total of 59 samples that included AA αα/αα (N = 10), AA − α/αα (N = 10), AA − α/− α (N = 11), AS αα/αα (N = 9), AS − α/αα (N = 9) & AS − α/− α (N = 10). Differences in rosette frequency and MFI by genotype were analyzed by linear regression and results are expressed as mean rosette frequency and MFI with bars representing 95% confidence intervals. The dotted line shows the mean rosetting frequency or MFI in the control (AA αα/αα) pRBCs. Rosette frequency and MFI in each sample were tested in duplicate.

Having observed an association between co-inheritance of HbAS and α^+^thalassemia with regard to cytoadhesion, we examined the effect of co-inheritance on rosetting, an important parasite virulence phenotype associated with severe malaria (Rowe et al., 1995, Rowe et al., 2009, Mercereau-Puijalon et al., 2008). For these experiments we used the rosetting parasite strains IT/R29 and TM284R + (Material and Methods) to investigate rosetting in a total of 59 and 91 mixed-genotype RBC samples respectively. We found no association between either heterozygous or homozygous α^+^thalassemia and rosette frequencies when using IT/R29 parasites (Figs. 3A, S5A & Table S1). However, rosetting was greatly reduced in HbAS pRBCs without α^+^thalassemia (mean frequency 10.2% (− 4.0–24.5) versus 56.0% (45.9–66.1) P < 0.001) (Figs. 3A, S5A & Table S1), an effect that was reversed in pRBCs of mixed HbAS and α^+^thalassemia genotype (lr test χ^2^ = 7.7, P = 0.021). These changes in rosette frequency were mirrored by changes in PfEMP1 expression levels in IT/R29 pRBCs, with PfEMP1 MFI and the proportion of ITvar9 positive pRBCs being reduced in HbAS but not α^+^thalassemia when considered individually (Figs. 3B, S5B, S5C & Table S1). The reduced PfEMP1 levels and the lower proportion of ITvar9 positive pRBCs seen in samples from HbAS subjects were reversed on co-inheritance with homozygous α^+^thalassemia (lr test χ^2^ = 16.95 (P < 0.001) and lr test χ^2^ = 9.75 (P = 0.008)) respectively (Figs. 3B, S5B, S5C & Table S1).

**Fig. 4:**
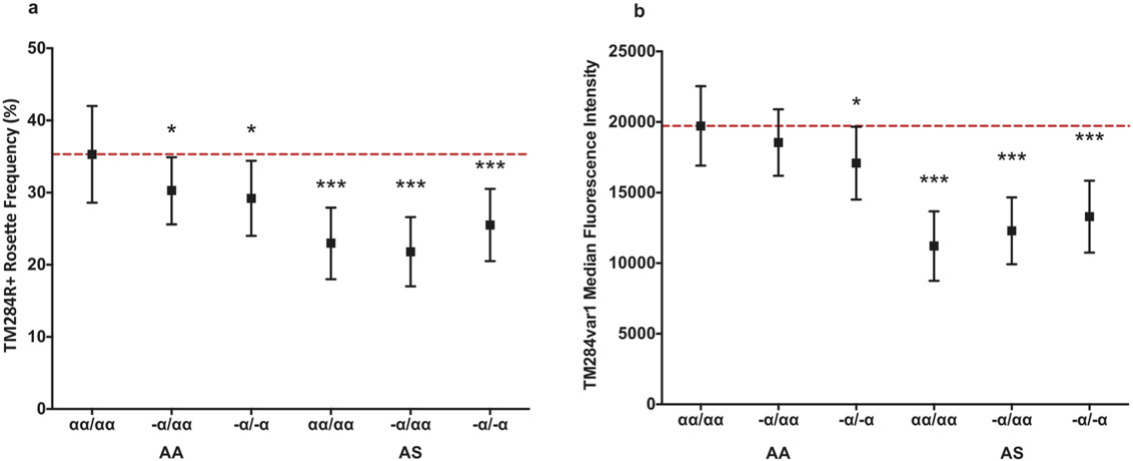
Rosette frequency and PfEMP1 expression in *P. falciparum* TM284R + pRBCs by HbAS and α^+^thalassemia genotype. (A) TM284R + rosette frequency. (B) TM284var1 PfEMP1 expression. Rosetting and PfEMP1 expression were assessed on a total of 91 samples (AA αα/αα (N = 18), AA − α/αα (N = 17), AA − α/− α (N = 16), AS αα/αα (N = 13), AS − α/αα (N = 15), AS − α/− α (N = 12)). Differences in rosette frequency and MFI by genotype were analyzed by linear regression with adjustment for CR1 RBC expression levels and additionally for the Sl Knops blood group when looking at rosette frequency. Results are expressed as the mean rosette frequency and MFI with bars representing 95% confidence intervals. The dotted line represents the mean rosetting frequency or MFI in the control (AA αα/αα) pRBCs. Rosette frequency and MFI in each sample were tested in duplicate in two independent experiments.

In experiments with a second *P. falciparum* rosetting strain TM284R +, rosetting and PfEMP1 expression showed a modest reduction in pRBCs with α^+^thalassemia alone and a more marked reduction in pRBCs with HbAS alone (Figs. 4, S6 & Table S1). As with IT/R29, the effect of HbS was reversed in pRBCs of mixed HbAS and α^+^thalassemia genotype for both rosetting (lr test χ^2^ = 6.70, P = 0.035) and PfEMP1 expression level (lr test χ^2^ = 7.34, P = 0.025) (Figs. 4, S6 & Table S1).

### 3.4 α^+^thalassemia is Associated With Reduced Proportions of Circulating HbS in HbAS Individuals in Kilifi

**Fig. 5:**
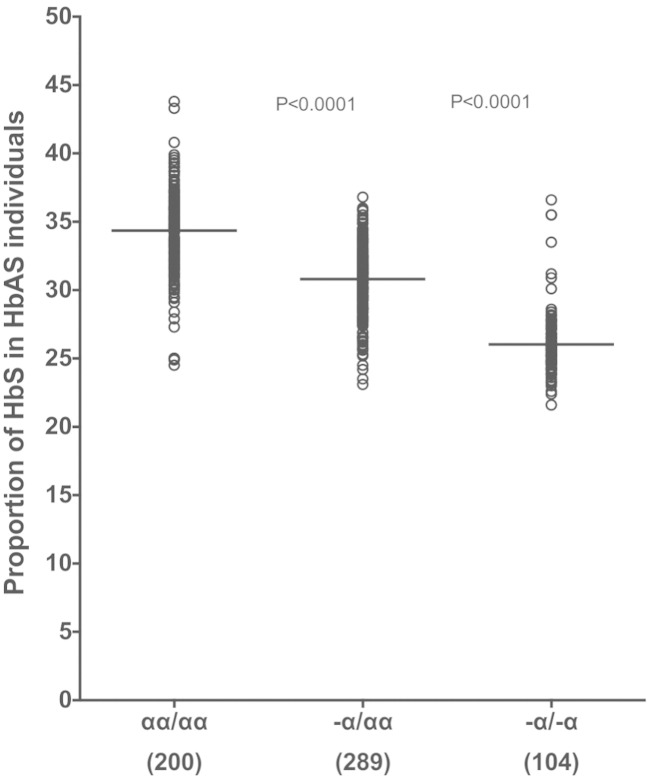
The association between α^+^thalassemia genotype and intracellular HbS proportion in Kenyan children. Proportion of HbS by α^+^thalassemia status was determined by HPLC on EDTA-anticoagulated blood collected from a total of 593 HbAS children aged between 9–12 months from the Kilifi Genetic Birth Cohort (KGBC) study. Lines represent mean HbS proportions. Differences in mean HbS proportions by genotype were tested using the ANOVA test with Holm Sidak's multiple comparisons post-hoc test. In parenthesis are number of samples for each genotype tested.

Previous studies in India and the Democratic Republic of Congo have shown that α^+^thalassemia is associated with a dose-dependent decrease in the proportion of total Hb that is represented by HbS in the RBCs of HbAS individuals (Brittenham et al., 1977, Mouele et al., 2000), a phenomenon that might play a part in the loss of malaria protection in HbAS subjects with co-inherited α^+^thalassemia (Hood et al., 1996). We investigated whether the same relationship between HbS proportion and α^+^thalassemia existed among HbAS subjects in our study population in Kilifi, Kenya. We used HPLC to measure the relative proportions of the various forms of hemoglobin (predominantly HbA, HbS and HbF) in whole blood samples collected from a cohort of 593 children with HbAS (see Materials and Methods). Compared with HbAS children without α^+^thalassemia (mean HbS proportion 34.3%), HbS proportions were significantly lower in α^+^thalassemia heterozygotes (30.8; mean difference, 3.5; P < 0.0001) and homozygotes (26.0; 8.3; P < 0.0001) (Fig. 5).

## 4 Discussion

A survival advantage against severe malaria is now widely accepted as the explanation for the occurrence at high frequencies of both HbAS and α^+^thalassemia in malaria-endemic populations. Nevertheless, little is known about the epidemiological and clinical consequences of the co-inheritance of these two conditions, a common occurrence in many parts of the malaria-endemic world. Recent studies from both the Kenyan Coast and from West Africa have provided evidence for a negative epistatic interaction between HbAS and α^+^thalassemia with regard to their protective effects against clinical malaria (Williams et al., 2005c, May et al., 2007, Crompton et al., 2008), a phenomenon that may explain differences in their distribution in various parts of the world (Penman et al., 2009, Penman et al., 2011).

In the present study, we explored the effects of coinheritance of HbAS and α^+^thalassemia on a range of phenotypes that are believed to be associated with the pathogenesis of severe and complicated malaria, including cytoadhesion, rosetting and PfEMP1 expression. We found that pRBCs from donors with HbAS showed reductions in cytoadhesion, rosetting and PfEMP1 expression in experiments involving three different *P. falciparum* strains and more than 300 RBC donors. Our data support those obtained in a previous study reported by Cholera and colleagues (Cholera et al., 2008) suggesting that HbAS might protect against severe malaria by reducing *P. falciparum* sequestration. However, we show for the first time that co-inheritance of HbAS with α^+^thalassemia reverses these effects such that pRBCs from donors with both mutations show levels of cytoadhesion, rosetting and PfEMP1 expression that are similar to those seen in normal pRBCs. Our data therefore support the hypothesis that the negative epistasis between HbAS and α^+^thalassemia observed in epidemiological studies might be explained by changes in the adhesion properties of *P. falciparum*-infected pRBCs.

The mechanisms by which α^+^thalassemia mutations reverse the effect of HbAS on PfEMP1 expression and pRBC adhesion are unclear. We have previously proposed (Williams et al., 2005c) that it might relate to the observation that concentrations of HbS in the RBCs of HbAS individuals are negatively correlated with the number of α-globin deletions, the lowest concentrations being seen in individuals with homozygous α^+^thalassemia (Mouele et al., 2000, Brittenham et al., 1980), a phenomenon that we have confirmed in the current study. Experiments conducted in mice with *Plasmodium yoelii*, a murine model for parasite sequestration, seem to support such a hypothesis: in one study, while transgenic mice expressing high HbS proportions developed low parasitemia infections and recovered completely, mice expressing lower levels of HbS experienced severe infections and died (Hood et al., 1996). Recently, it has been suggested that HbS might interfere with the development of the parasite trafficking pathways that are involved in the transportation of PfEMP1 and other molecules to the pRBC surface (Cyrklaff et al., 2011, Kilian et al., 2013). It seems plausible that this trafficking defect might be critically dependent on the intracellular concentration of HbS, providing a mechanistic link between intracellular HbS concentration and cell surface PfEMP1 expression levels that can be tested in future work.

One issue that was not resolved in the current study is the mechanism by which α^+^thalassemia on its own results in protection from severe malaria. Although a previous study reported both reduced binding to human microvascular endothelial cells and reduced PfEMP1 expression in α^+^thalassemic pRBCs (Krause et al., 2012), this finding contrasts with other previous studies (Williams et al., 2002, Luzzi et al., 1991a, Luzzi et al., 1991b), and was also not supported by our current study, in which we saw reduced cytoadhesion (Fig. 1), but no concomitant reduction in PfEMP1 expression (Fig. 2). The differences between our findings and those of Krause and colleagues may well reflect a difference in experimental design. While Krause and colleagues used multiple *P. falciparum* strains in their experiments, they only included 4–5 donor controls and between 2 and 6 RBC donors of each variant genotype. Furthermore, their PfEMP1 experiments included no individuals with homozygous α^+^thalassemia. Data from our current and a previous study (Williams et al., 2002) both show that even when using a single *P. falciparum* strain, there is considerable variation in the levels of cytoadhesion and PfEMP1 expression between different donors of the same genotype (Figs. S3 and S4). It is possible that studies using small numbers of RBC donors might produce donor-specific results that may not be truly representative. For this reason, we used a reductionist approach in our current study, keeping the parasite strain constant and using large numbers of RBC donors within each experiment. However, our current data do suggest that the parasite strain used may influence the results seen in studies of α^+^thalassemic RBCs. For example, the rosetting parasite TM284R + showed a minor reduction in rosette frequency in α^+^thalassemic RBCs whereas the IT/R29 parasite did not (Fig. 3, Fig. 4). Previous work has suggested reduced rosetting in α^+^thalassemic RBCs (Carlson et al., 1994, Udomsangpetch et al., 1993a); however, these studies also suffer from limitations in the samples tested. Carlson et al. (1994) studied one parasite strain with three α^+^thalassemia heterozygote donors, while Udomsangpetch et al. (1993a) studied one parasite strain and a heterogeneous group of 20 donors that included more severe hematological abnormalities such as Hb Constant Spring and HbE. Taken together, the above studies show that the effect of α^+^thalassemia on pRBC adhesion and PfEMP1 expression remains unresolved, possibly due to parasite strain-specific effects and differences in experimental design between existing studies. Future studies of this sort should aim to maximize both the number of donors and the number of *P. falciparum* strains within technically feasible limits.

In the current study we investigated PfEMP1 expression in RBCs that had been previously cryopreserved and thawed prior to inoculation with *P. falciparum*. While it is possible that this approach could lead to genotype-specific artifacts, data from our laboratory using fresh RBCs and a different ICAM1 and CD36 binding laboratory-adapted parasite strain, A4U, also showed reduced PfEMP1 expression in HbS pRBC that was reversed with co-inheritance of α^+^thalassemia (supplementary material, Fig. S7). Therefore a consistent effect of RBC genotype on PfEMP1 expression is seen with four different *P. falciparum* strains, using both fresh and cryopreserved cells.

In conclusion, our data suggest that the negative epistatic interaction between HbAS and α^+^thalassemia with regard to malaria protection that is seen at an epidemiological level might in part be explained by changes in the cytoadhesion and rosetting properties of mixed-genotype pRBCs, which might in turn relate to altered expression of PfEMP1. It remains to be tested how this relates to defects in parasite protein trafficking systems (Cyrklaff et al., 2011), and whether other important mechanisms of protection including altered knob expression on the surface of pRBCs (Cholera et al., 2008, Krause et al., 2012), enhanced immune responses (Williams et al., 2005d, Verra et al., 2007) and phagocytosis (Ayi et al., 2004) might also be attenuated in this negative interaction.

## Funding

This work was funded by the Wellcome Trust through Senior Research Fellowships awarded to TNW (grant no. 091758) and JAR (grant no. 084226), through core support to the KEMRI-Wellcome Trust Programme (grant no. 084535) and through a sub-grant from a Wellcome Trust Strategic Award (grant no. 084538) to DHO.

## Role of the Funding Source

The funders played no role in the design of the study, data collection and analysis, the decision to publish or in the preparation of the manuscript.

## Author Contributions

DHO, LBO, DJPF, JAR, and TNW designed the research; DHO, LBO, MT, BRS, HO, HF, and DJPF performed the research; AG, and DJPF contributed new reagents/analytic tools; DHO, DJPF, JAR, and TNW analyzed the data; and DHO, LBO, DJPF, JAR and TNW wrote the paper.

## Conflict of Interest

The authors have declared that no conflicting interests exist.
