# Supplementary material for: Mechanistic Studies of the Negative Epistatic Malaria-protective Interaction Between Sickle Cell Trait and α+thalassemia
Source: eBioMedicine. 2014 Oct 13;1(1):29–36. doi: 10.1016/j.ebiom.2014.10.006 (PMC4397954; doi:10.1016/j.ebiom.2014.10.006)
Supplement: Supplementary file 1 — Supplementary material. [file mmc1.doc]

**Supplementary information**

**Supplementary Tables**

**Table S1. Cytoadherence, rosetting and PfEMP1 expression by HbAS and α^+^**thalassemia genotypes

| **ItG static adhesion to CD36** | | | | | |
| --- | --- | --- | --- | --- | --- |
|  |  | **Mean pRBCs bound/mm^2^** | **95% CI** | **P value** | **LR χ^2^, P value** |
| AA | αα/αα | 1913.4 | 1329.1 – 2603.9 | - | 11.14, 0.004 |
|  | -α/αα | 1543.6 | 1055.8 – 2123.8 | 0.196 |  |
|  | -α/-α | 1001.7 | 635.9 – 1450.2 | <0.001 |  |
| AS | αα/αα | 1263.1 | 774.3 – 1870.8 | 0.037 |  |
|  | -α/αα | 1569.6 | 1084.8 – 2143.6 | 0.223 |  |
|  | -α/-α | 1755.8 | 1127.9 – 2522.0 | 0.661 |  |
| **ItG static adhesion to ICAM1** | | | | | |
|  |  | **Mean pRBCs bound/mm^2^** | **95% CI** | **P value** | **LR χ^2^, P value** |
| AA | αα/αα | 2858.2 | 2057.1 – 3790.8 | - | 7.50, 0.024 |
|  | -α/αα | 2334.6 | 1661.0 – 3122.5 | 0.180 |  |
|  | -α/-α | 1891.9 | 1320.5 – 2565.7 | 0.007 |  |
| AS | αα/αα | 1819.2 | 1160.7 – 2625.1 | 0.014 |  |
|  | -α/αα | 2272.5 | 1618.9 – 3036.6 | 0.125 |  |
|  | -α/-α | 2404.3 | 1582.4 – 3397.6 | 0.344 |  |
| **ItG (ITvar16) PfEMP1 expression** | | | | | |
|  |  | **Mean MFI** | **95% CI** | **P value** | **LR χ^2^, P value** |
| AA | αα/αα | 346.2 | 271.0 – 421.3 | - | 0.47, 0.789 |
|  | -α/αα | 389.9 | 307.8 – 472.0 | 0.290 |  |
|  | -α/-α | 469.0 | 388.0 – 549.9 | 0.004 |  |
| AS | αα/αα | 134.6 | 68.7 – 200.5 | <0.001 |  |
|  | -α/αα | 178.4 | 72.3 – 284.4 | 0.002 |  |
|  | -α/-α | 257.4 | 152.6 – 362.2 | 0.095 |  |
| **IT/R29 rosetting** | | | | | |
|  |  | **Mean rosette frequency** | **95% CI** | **P value** | **LR χ^2^, P value** |
| AA | αα/αα | 56.0 | 45.9 – 66.1 | - | 7.7, 0.021 |
|  | -α/αα | 59.8 | 45.8 – 73.7 | 0.588 |  |
|  | -α/-α | 58.3 | 44.5 – 72.0 | 0.739 |  |
| AS | αα/αα | 10.2 | -4.0 – 24.5 | <0.001 |  |
|  | -α/αα | 19.7 | 5.4 – 34.0 | <0.001 |  |
|  | -α/-α | 37.2 | 22.3 – 52.0 | 0.014 |  |
| **IT/R29 (ITvar9) PfEMP1 expression** | | | | | |
|  |  | **Mean MFI** | **95% CI** | **P value** | **LR χ^2^, P value** |
| AA | αα/αα | 3495.4 | 3141.9 – 3849.0 | - | 16.95, <0.001 |
|  | -α/αα | 3801.7 | 3312.2 – 4291.2 | 0.215 |  |
|  | -α/-α | 4135.9 | 3654.4 – 4617.4 | 0.010 |  |
| AS | αα/αα | 1711.7 | 1212.2 – 2211.3 | <0.001 |  |
|  | -α/αα | 2157.7 | 1658.2 – 2657.2 | <0.001 |  |
|  | -α/-α | 3627.6 | 3107.4 – 4147.8 | 0.612 |  |
| **Percentage ITvar9 PfEMP1 expression** | | | | | |
|  |  | **% pRBCs ITvar9 positive** | **95% CI** | **P value** | **LR χ^2^, P value** |
| AA | αα/αα | 58.6 | 54.2 – 62.9 | - | 9.75, 0.008 |
|  | -α/αα | 57.2 | 51.2 – 63.3 | 0.662 |  |
|  | -α/-α | 60.3 | 54.4 – 66.3 | 0.554 |  |
| AS | αα/αα | 41.4 | 35.2 – 47.6 | <0.001 |  |
|  | -α/αα | 50.6 | 44.4 – 56.8 | 0.013 |  |
|  | -α/-α | 55.0 | 48.6 – 61.5 | 0.276 |  |
| **TM284R+ rosetting** | | | | | |
|  |  | **Mean rosette frequency** | **95% CI** | **P value** | **LR χ^2^, P value** |
| AA | αα/αα | 35.3 | 28.6 – 42.0 | - | 6.70, 0.035 |
|  | -α/αα | 30.3 | 25.6 – 34.9 | 0.033 |  |
|  | -α/-α | 29.2 | 24.0 – 34.4 | 0.021 |  |
| AS | αα/αα | 23.0 | 18.0 – 27.9 | <0.001 |  |
|  | -α/αα | 21.8 | 17.0 – 26.6 | <0.001 |  |
|  | -α/-α | 25.5 | 20.5 – 30.5 | <0.001 |  |
| **TM284R+ PfEMP1 expression** | | | | | |
|  |  | **Mean MFI** | **95% CI** | **P value** | **LR χ^2^, P value** |
| AA | αα/αα | 19721.6 | 16905.5 – 22537.6 | - | 7.34, 0.025 |
|  | -α/αα | 18548.0 | 16194.5 – 20901.6 | 0.324 |  |
|  | -α/-α | 17087.2 | 14507.3 – 19667.1 | 0.045 |  |
| AS | αα/αα | 11213.8 | 8750.7 – 13677.0 | <0.001 |  |
|  | -α/αα | 12292.7 | 9922.1 – 14663.4 | <0.001 |  |
|  | -α/-α | 13294.2 | 10748.6 – 15839.8 | <0.001 |  |
| **Percentage TM284var1 PfEMP1 expression** | | | | | |
|  |  | **% pRBCs TM284var1 positive** | **95% CI** | **P value** | **LR χ^2^, P value** |
| AA | αα/αα | 40.8 | 37.7 – 43.9 | - | 0.32, 0.853 |
|  | -α/αα | 37.8 | 34.8 – 40.9 | 0.054 |  |
|  | -α/-α | 38.6 | 35.4 – 4175 | 0.160 |  |
| AS | αα/αα | 36.6 | 34.0 – 39.2 | 0.002 |  |
|  | -α/αα | 33.6 | 29.7 – 37.5 | <0.001 |  |
|  | -α/-α | 34.3 | 30.3 – 38.4 | 0.002 |  |

**Figure legends**

**Figure S1. Gating strategy for ITvar16 PfEMP1 expression**

ITvar16 PfEMP1 expression was determined by staining live pRBCs with Hoechst 33342 (to detect the pRBC population), rat polyclonal antisera raised against the ITvar16 PfEMP1 variant, goat anti-rat IgG and a tertiary incubation with Alexa Fluor 488 conjugated donkey anti-goat IgG. A) An example of an ItG negative control sample (no primary antibody), showing Hoechst-staining of the pRBC population (right half), and some background staining with Alexa Fluor 488 on both uninfected and pRBCs. B) An example of an ItG positive sample incubated with ITvar16 polyclonal rat sera, showing a shift in Alexa Fluor 488 staining of the pRBC population (right half) compared to the negative control. Specific ITvar16 PfEMP1 expression was determined by subtracting the Alexa Fluor 488 Median Fluorescence Intensity (MFI) of the pRBC population in the negative control from the equivalent value in the positive sample.

**Figure S2. Gating strategy for ITvar9, TM284var1 and A4var PfEMP1 expression**

For the determination of ITvar9 and TM284var1 PfEMP1 expression, live pRBCs were stained with Hoechst 33342 (to detect the pRBC population), anti-ITvar9 or anti-TM284var1 rabbit polyclonal total IgG respectively followed by a secondary antibody staining with Alexa 488 conjugated goat anti-rabbit IgG. The staining protocol for A4var is shown in the legend for Fig S7. Similar gating strategies were used for ITvar9, TM284var1 and A4var, and ITvar9 is shown as an example. A) A negative control sample stained with 10 μg/ml total IgG from a non-immunised rabbit shows the Hoechst positive pRBC population (right lower quadrant). B) A test sample incubated with 10 μg/ml anti-ITvar9 rabbit polyclonal IgG shows positive pRBCs (Alexa 488 positive and Hoechst positive) in the upper right quadrant, and the Alexa 488 Median Fluorescent Intensity (MFI) was determined on this population. Some pRBCs are negative for ITvar9 staining (lower right quadrant) because they express different PfEMP1 variants. Using the positive samples, the ITvar9-specific MFI was determined by subtracting from the ITvar9 positive pRBC MFI (upper right quadrant) the MFI of the uninfected RBCs (lower left quadrant), to adjust for any non-specific background staining. The proportion of pRBCs positive for ITvar9 was determined by subtracting the proportion of pRBCs positively staining for ITvar9 in the negative rabbit IgG samples (upper right quadrant) from similar values in the positive samples.

**Figure S3. Relative cytoadherence of *P. falciparum* ItG pRBCs by HbS and α^+^thalassemia genotype.**

A) Relative binding to CD36 recombinant protein. B) Relative binding to ICAM-1 recombinant protein. Static adhesion to immobilized proteins on plastic was tested in a total of 99 RBC samples representing the six possible HbAS and α^+^thalassemia genotype combinations: AA αα/αα (N=21), AA -α/αα (N=18), AA -α/-α (N=21), AS αα/αα (N=11), AS -α/αα (N=18) & AS -α/-α (N=10). Samples were tested over five experimental days (day 1 n=13, day 2 n=8, day 3 n=55, day 4 n=15, day 5 n=8). For each day, the binding data for each sample were normalized to that of the mean binding for the control pRBCs (AA αα/αα) run on the same day. For each RBC sample, adhesion was tested in two dishes with triplicate protein spots in each dish. Horizontal bars represent median relative adhesion for each genotype. P values <0.05 are shown (Kruskal-Wallis test with Dunn’s post-hoc multiple comparisons test).

**Figure S4. PfEMP1 expression in *P. falciparum* ItG pRBCs by HbS and α^+^thalassemia genotype.**

PfEMP1 expression was tested by flow cytometry using rat polyclonal antisera against the ITvar16 PfEMP1 variant predominantly expressed by the ItG parasite strain. A total of 60 RBC samples representing a range of genotype combinations were tested: AA αα/αα (N=10), AA -α/αα (N=10), AA -α/-α (N=11), AS αα/αα (N=10), AS -α/αα (N=9), AS -α/-α (N=10). All samples were tested on the same day, therefore raw data are shown without normalization. PfEMP1 expression was assessed by determining the specific Alexa 488 median fluorescent intensity (MFI) of pRBCs (see methods). Horizontal bars represent mean MFI for each genotype. P values <0.05 are shown (Kruskal-Wallis test with Dunn’s post-hoc multiple comparisons test).

**Figure S5. Relative rosette frequency and PfEMP1 expression in *P. falciparum* IT/R29 pRBCs by HbS and α^+^thalassemia genotype.**

(A) Relative IT/R29 rosette frequency. (B) Relative ITvar9 PfEMP1 expression. (C) Relative ITvar9 positive pRBCs. Rosetting and ITvar9 PfEMP1 expression in the IT/R29 parasite strain were assessed in a total of 59 samples: AA αα/αα (N=10), AA -α/αα (N=10), AA -α/-α (N=11), AS αα/αα (N=9), AS -α/αα (N=9), AS -α/-α (N=10). Samples were tested over two consecutive experimental days (day 1=30 and day 2=29). Rosette frequency, MFI and proportion of ITvar9 positive pRBCs data for all samples were normalized to that of the mean rosette frequency, MFI and proportion of ITvar9 positive pRBCs for the control pRBCs (AA αα/αα) run on the same day. Horizontal bars represent median relative rosette frequency, MFI and proportion of ITvar9 positive pRBCs for each genotype. P values <0.05 are shown (Kruskal-Wallis test with Dunn’s post-hoc multiple comparisons test).

**Figure S6. Relative rosette frequency and PfEMP1 expression in *P. falciparum* TM284R+ pRBCs by HbS and α^+^thalassemia genotype.**

(A) Relative TM284R+ rosette frequency. (B) Relative TM284var1 PfEMP1 expression. (C) Relative TM284R+ positive pRBCs. Rosetting and TM284var1 PfEMP1 expression were assessed in a total of 91 samples: AA αα/αα (N=18), AA -α/αα (N=17), AA -α/-α (N=16), AS αα/αα (N=13), AS -α/αα (N=15) & AS -α/-α (N=12). The samples were tested over three experiments divided by ABO blood groups, O (n=31), A (n=29) and B and AB (n=31), each involving two independent experiments, with each sample tested in duplicate. Rosette frequency, MFI and proportion of TM284R+ positive pRBCs data for all samples were normalized to that of the mean rosette frequency, MFI and proportion of TM284R+ positive pRBCs for the control pRBCs (AA αα/αα) run on the same day. Horizontal bars represent the median relative rosette frequency, MFI and proportion of TM284R+ positive pRBCs for each genotype. P values <0.05 are shown (Kruskal-Wallis test with Dunn’s post-hoc multiple comparisons test).

**Figure S7: Relative PfEMP1 expression tested in fresh RBCs infected with *P. falciparum* A4U parasites by HbS and α^+^thalassemia genotype.**

PfEMP1 expression in all other parasite lines used in this study was tested using RBC samples that had been cryopreserved and thawed prior to inoculation with *P. falciparum* parasites. To test whether the results seen with cryopreserved RBCs are similar to those seen with fresh RBCs, we tested for PfEMP1 expression using A4U parasites in fresh RBC samples, used within four days of collection. A4U is from the same parental genotype, IT4/25/5, as ItG, and similarly binds to both CD36 and ICAM1[1). A4U parasites predominantly express the PfEMP1 variant encoded by the *A4var* gene (also known as ITvar14) (2). A total of 142 RBC samples including (AA αα/αα (N=24), AA -α/αα (N=28), AA -α/-α (N=29), AS αα/αα (N=20), AS -α/αα (N=21) & AS -α/-α (N=20)) were inoculated with magnetically purified pigmented trophozoite-stage A4U parasites to give a starting parasitemia of 1.5%. A4var PfEMP1 expression was then determined by flow cytometry on mature pigmented trophozoites after one complete parasite life cycle, by staining with 0.5 μg/ml of the BC6 mouse mAb which specifically recognizes the A4var PfEMP1 variant (3) (gift from Prof. Chris Newbold, University of Oxford). After washing the cells, this was followed by incubation with 1:50 dilution of rabbit anti-mouse IgG (STAR26B – AbD Serotec), 1:100 dilution of Alexa 488 conjugated goat anti-rabbit IgG (H+L) (Invitrogen) and 10 μg/ml of ethidium bromide to stain parasite populations before analysis on a FC500 flow cytometer (Beckman, coulter). A total of 100,000 events were acquired for each sample. These experiments were carried out over six experimental days and the results are presented in two ways. a) Differences in A4var PfEMP1 expression by genotype were analyzed by linear regression with adjustment for confounding by the ABO blood group and experimental day. Results are expressed as the mean MFI (95% CI). The dotted line shows the mean MFI in the control (AA αα/αα) pRBCs. MFI for each sample was tested in duplicate. * p<0.05. b) MFI data for all samples were normalized to that of the mean MFI for the control pRBCs (AA αα/αα) run on the same day. Data are presented this way to display variation that exists in PfEMP1 expression in donors within each genotype. Horizontal bars represent the median MFI for each genotype. Comparisons in PfEMP1 expression between genotypes were done using the non-parametric Kruskal-Wallis test with Dunn’s post-hoc multiple comparisons test.

**Supplementary references:**

1. Roberts DJ, Craig AG, Berendt AR, Pinches R, Nash G, Marsh K, Newbold CI (1992). Rapid switching to multiple antigenic and adhesive phenotypes in malaria. Nature 357:689-692.

2. Smith JD, Kyes S, Craig AG, Fagan T, Hudson-Taylor D, Miller LH, Baruch DI, Newbold CI (1998). Analysis of adhesive domains from the A4VAR *Plasmodium falciparum* erythrocyte membrane protein-1 identifies a CD36 binding domain. Mol Biochem Parasitol 97:133-148.

3. Smith JD, Chitnis CE, Craig AG, Roberts DJ, Hudson-Taylor DE, Peterson DS, Pinches R, Newbold CI, Miller LH (1995). Switches in expression of *Plasmodium falciparum var* genes correlate with changes in antigenic and cytoadherent phenotypes of infected erythrocytes. Cell 82:101-110.

**Figure S1.**

**
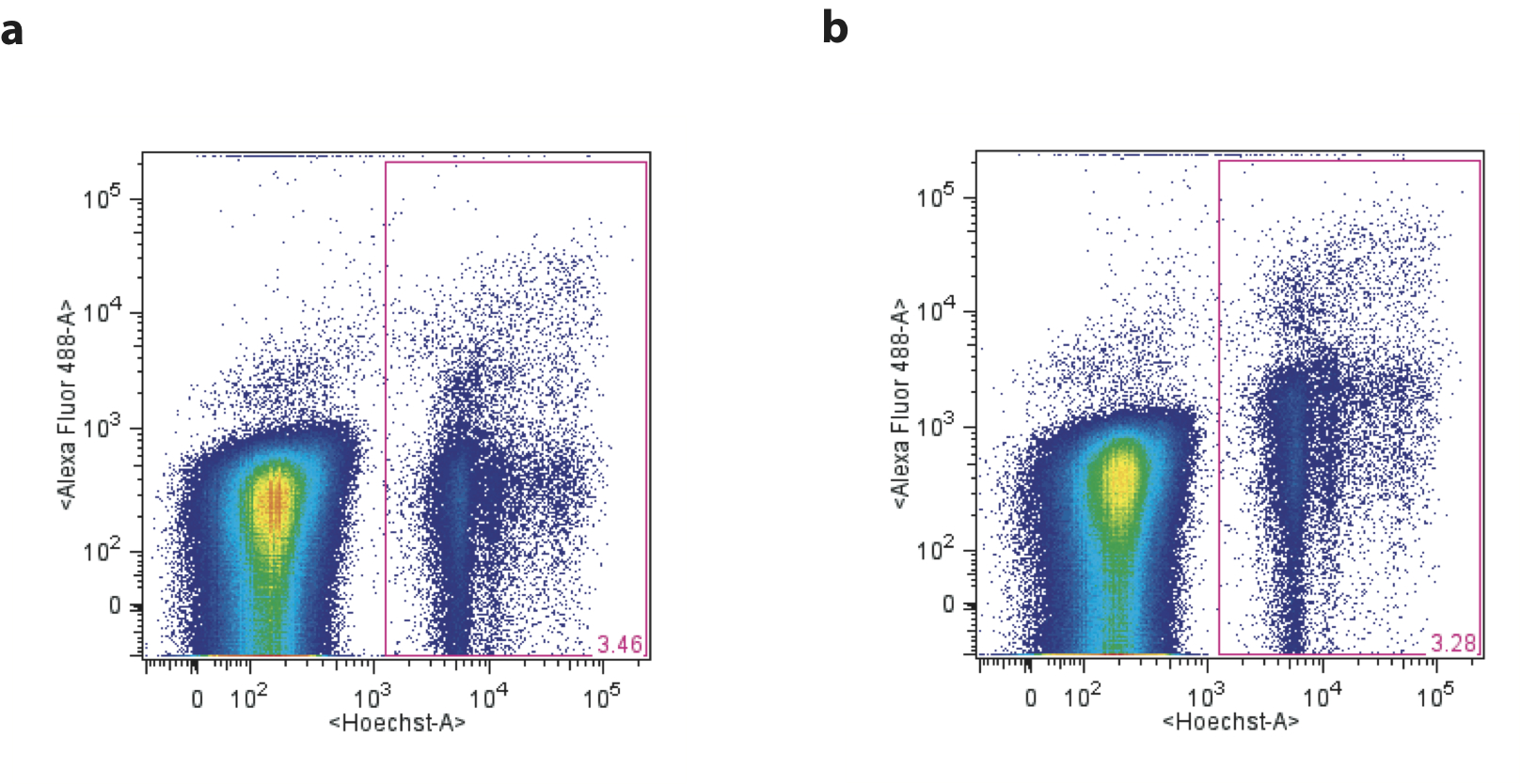
**

**Figure S2.**

**
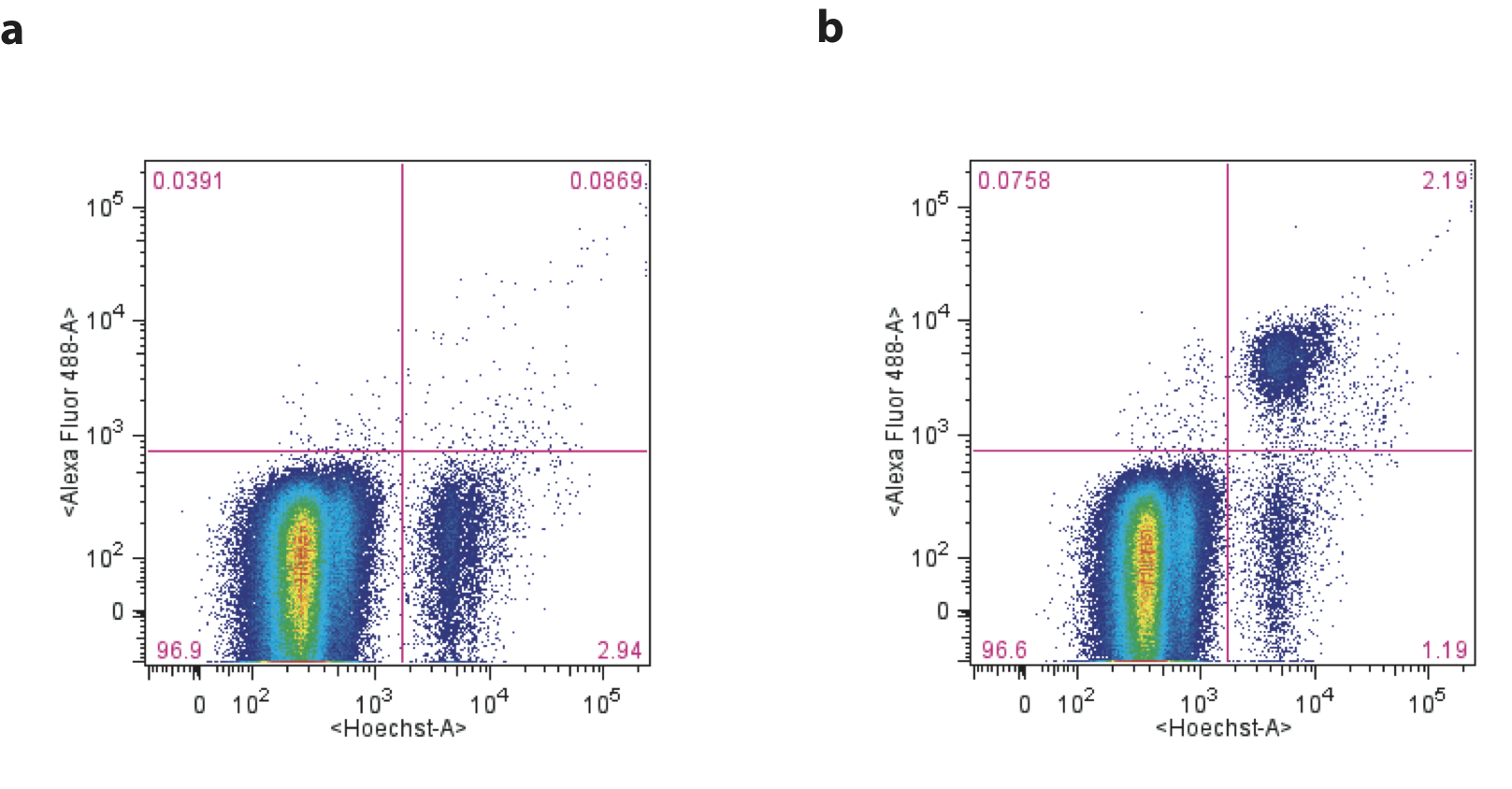
**

**Figure S3.**


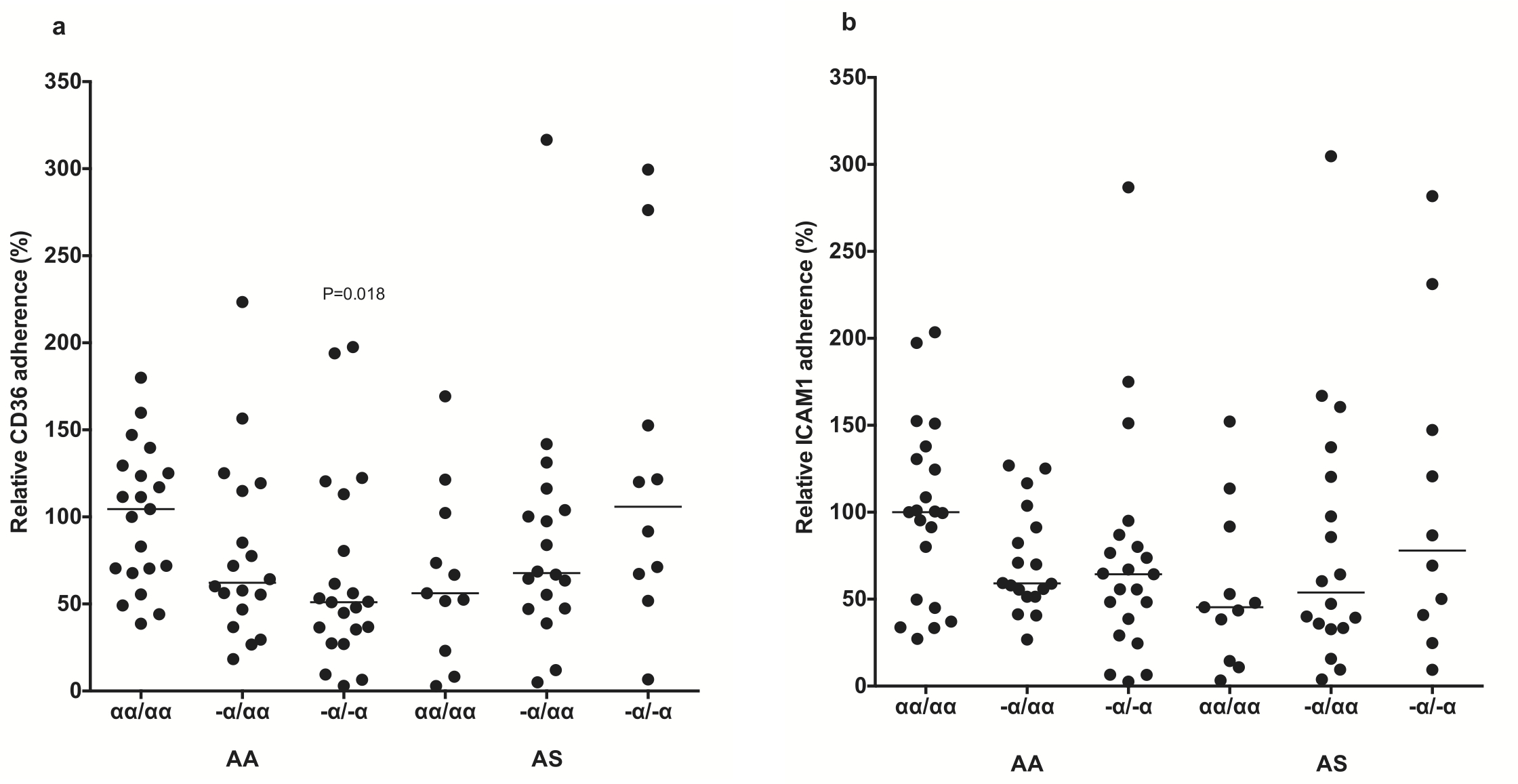


**Figure S4.**

**
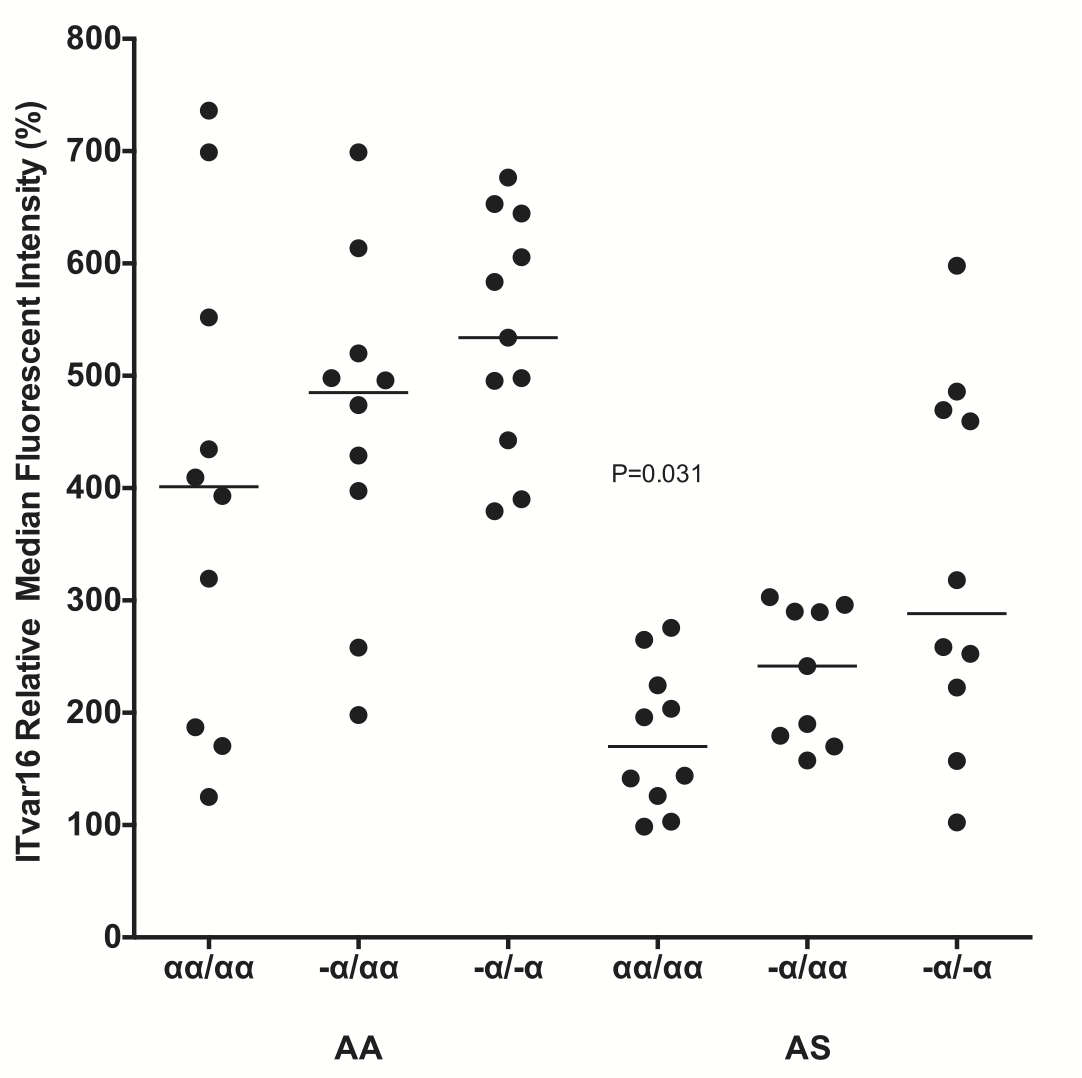
**

**Figure S5.**


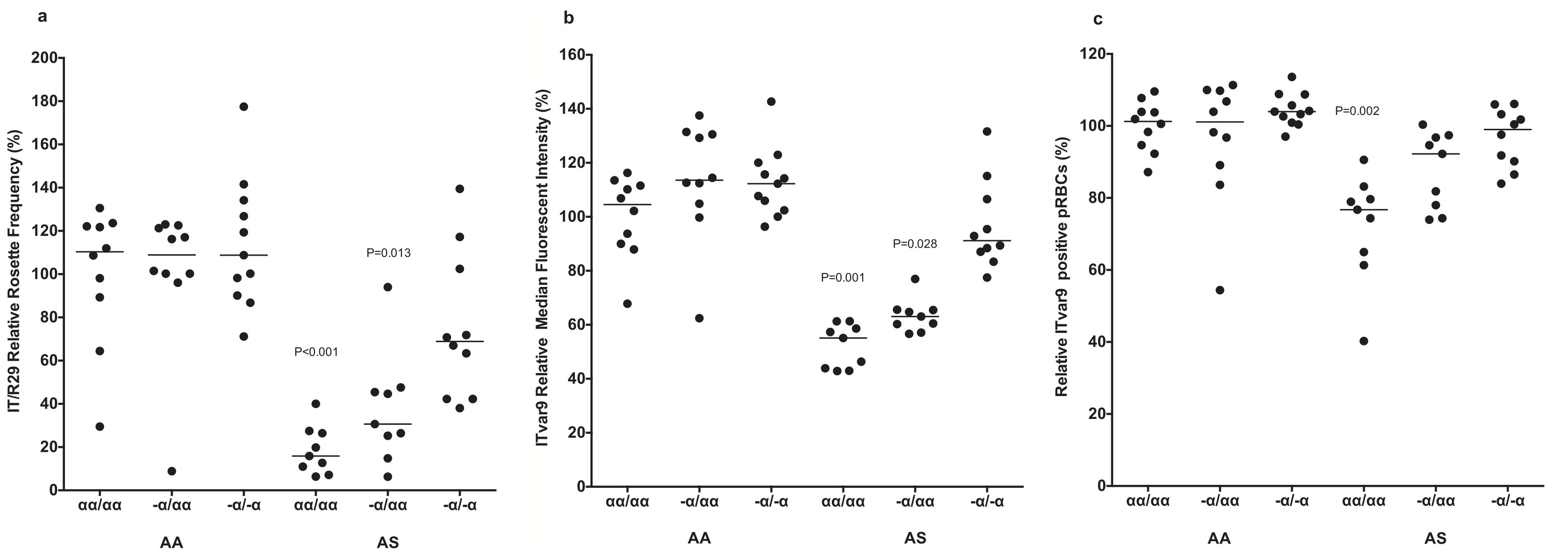


**Figure S6.**

**
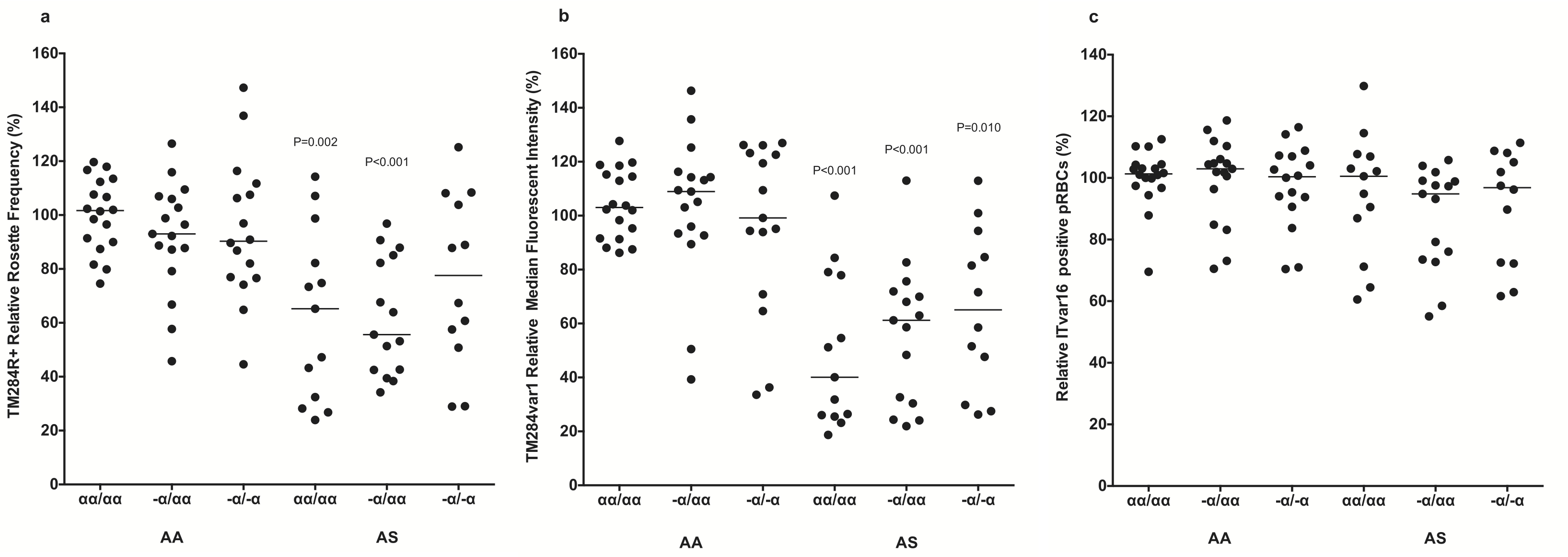
**

**Figure S7**

**
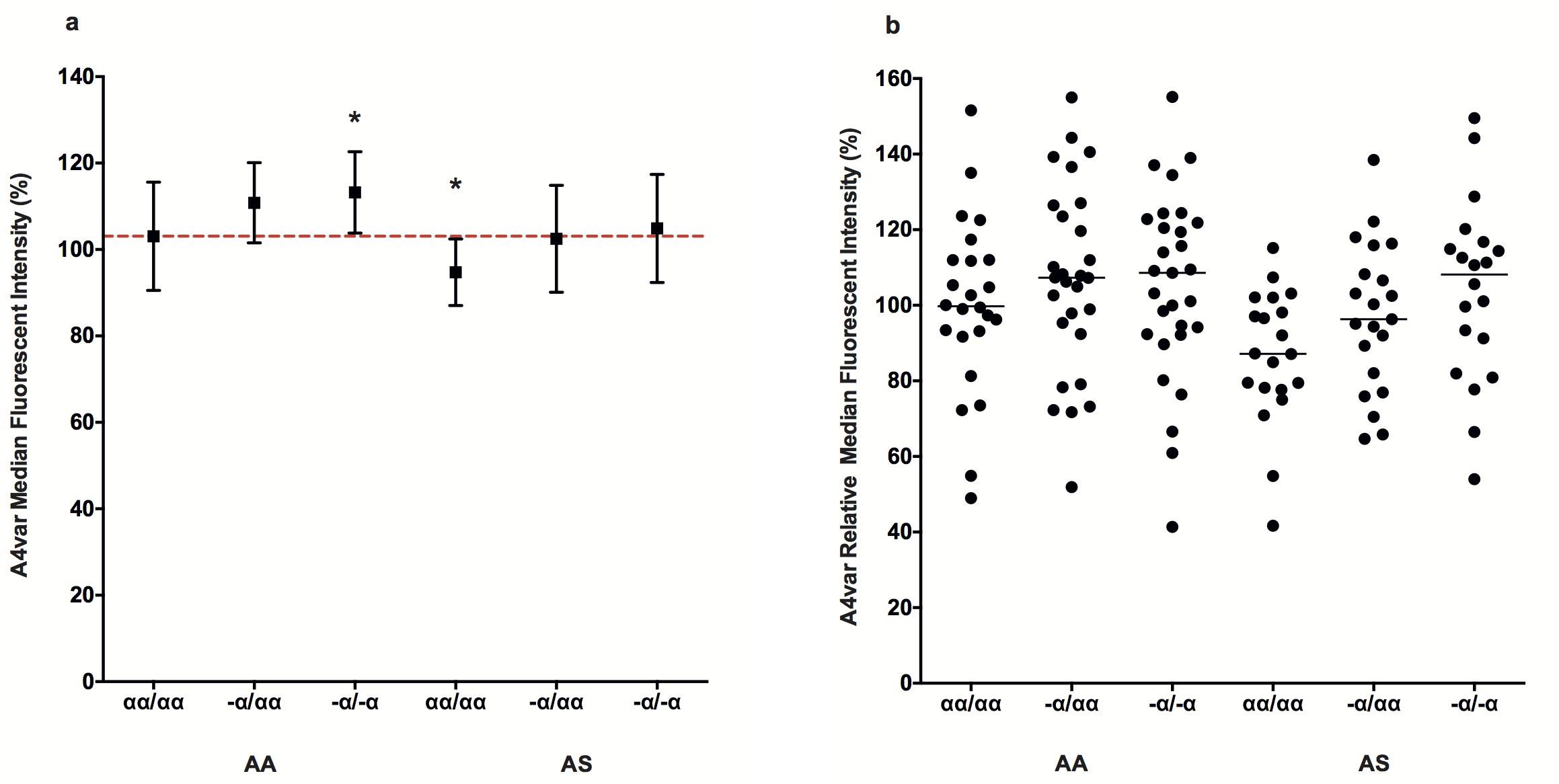
**
